# Application of the transoral endoscopic vestibular approach for a benign upper neck mass: A case report

**DOI:** 10.1097/MD.0000000000024087

**Published:** 2021-01-15

**Authors:** Dong Won Lee, Jeong Kyu Kim, Kyung Tae

**Affiliations:** a Department of Otorhinolaryngology – Head and Neck Surgery, School of Medicine, Catholic University of Daegu, Daegu; b Department of Otolaryngology – Head and Neck Surgery, College of Medicine, Hanyang University, Seoul, Republic of Korea.

**Keywords:** benign mass, NOTES, transoral neck surgery, transoral vestibular approach

## Abstract

**Rationale:**

Excision of a benign neck mass is traditionally performed via the transcervical approach. In order to avoid visible scars in the neck, various remote access surgical approaches have been developed. The aim of this report is to present the technique of a transoral endoscopic vestibular approach for treatment of a benign upper neck mass.

**Patient concerns:**

A 47-year-old female with an anterior upper neck mass and throat discomfort visited our institution.

**Diagnosis:**

The computed tomography (CT) scans and ultrasound (US) images demonstrated a benign-appearing mass on the anterior upper neck area.

**Interventions:**

The benign upper neck mass was successfully removed via the transoral endoscopic vestibular approach, without any complication.

**Outcomes:**

The final pathologic report indicated that the lesion was an epidermal cyst. The cosmetic result was excellent.

**Lessons:**

From this case study, we can learn to apply a transoral endoscopic vestibular approach for removal of a benign upper neck mass. Based on the outcomes, endoscopic removal of the benign upper neck mass via a transoral vestibular approach can be useful for patients who wish to hide any anterior neck scar.

## 1 Introduction

The primary treatment for a benign neck mass is surgical excision, which has traditionally been performed through a conventional transcervical approach.^[1]^ The transcervical approach is utilized in order to ensure a direct surgical field of view, to facilitate extraction of the mass, and to preserve the main anatomical neck structures. However, the procedure leaves an undesirable scar on the neck, which is the leading cause of the decrease in patient satisfaction following treatment. Therefore, various remote access and minimally invasive endoscopic or robotic neck surgery techniques have been developed to hide neck scars associated with surgery involving the thyroid, parathyroid, submandibular gland, and other neck masses.^[1–4]^

We have been performing endoscopic thyroidectomy via a transoral vestibular approach since 2017, and we have already reported regarding its feasibility and safety in thyroid surgery.^[5–9]^ The transoral vestibular approach is a promising procedure as a form of natural orifice transluminal endoscopic surgery (NOTES), which is truly scar-free, and less invasive than other types of remote access methods.^[10]^ For this patient, we applied this approach to remove her benign upper neck mass. We herein report her case in order to introduce a new, truly scarless technique for benign upper neck mass excision, and to comment on what we learned from it.

## 2 Case report

**Figure 1:**
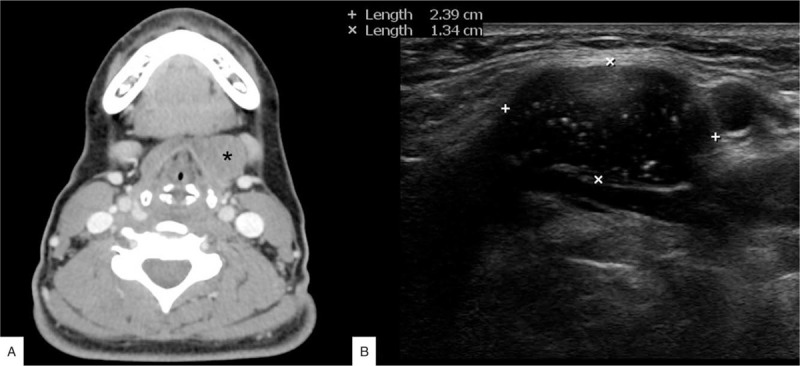
(A) Preoperative computed tomography (CT) scan. A 2.4 cm sized benign-appearing mass in the left infrahyoid region of the neck is noted, compressing the strap muscle (asterisk: mass). (B) Preoperative ultrasonographic findings. A thin-walled, heterogenous hypoechoic mass with internal hyperechoic debris is visualized.

Written informed consent was obtained from the patient and her family for the publication of the case. A 47-year-old female presented with a 10-year history of anterior upper neck mass and throat discomfort. She had no previous neck surgery or underlying disease. Neck computed tomography (CT) scan revealed a 2.4 cm benign-appearing mass in the left infrahyoid region of the neck, compressing the strap muscle (Fig. 1A). Fine needle aspiration biopsy (FNAB) demonstrated no malignant features. Ultrasonographic examination revealed a thin-walled, heterogeneous hypoechoic mass with internal hyperechoic debris (Fig. 1B). Findings were suggestive of a benign solid tumor with squamous epithelium lining. Under the impression of an epidermoid cyst, endoscopic excision via a transoral vestibular approach was performed.

## 3 Surgical technique

**Figure 2:**
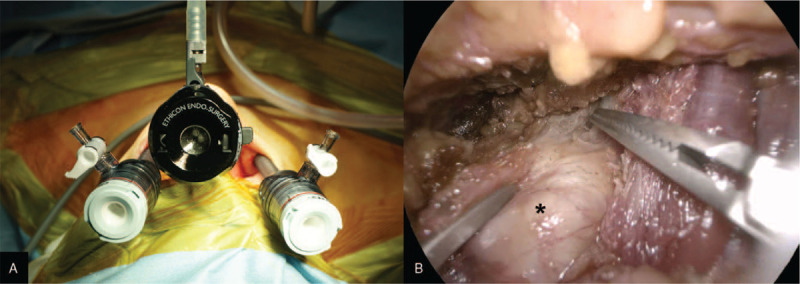
(A) Placement of the trocars. (B) Intraoperative endoscopic view. The whitish cystic mass was identified and was carefully dissected from the surrounding muscle fascia (asterisk: mass).

The patient was placed in the supine position on the operating table, under general anesthesia with orotracheal intubation. The neck was slightly extended using a shoulder pillow. The oral cavity was disinfected with povidone in normal saline. Skin preparation and draping were performed in the standard manner. A 2 cm horizontal incision was made from 1 cm above the frenulum of the lower lip. Twenty milliliter of normal saline with 0.05 mL of 0.1% epinephrine (1:400,000) was injected using a Veress needle syringe for hydrodissection via the central and lateral axes. The blunt dissection was done in the submental area using a Hegar dilator. A blunt-tipped 12 mm trocar was placed at the central incision site for a 10 mm 30-degree rigid endoscope. The CO_2_ insufflation pressure was maintained at 6 mm Hg. Two 5 mm trocars were inserted on either side of the endoscope in the oral vestibule for endoscopic instruments (Fig. 2A). The skin flap was elevated in the plane of the subplatysmal layer under the endoscopic view with laparoscopic dissectors, a hook monopolar electrocautery, and Harmonic curved shears. The dissection for the working space was widened to the level of the lower neck inferiorly, and the sternocleidomastoid muscle laterally. We did not use any external stitch for a better surgical view.

After creating the working space with a subplatysmal skin flap, the fascia overlying the mass was dissected to expose the mass using laparoscopic dissectors and Harmonic curved shears. The whitish cystic mass was identified, then carefully dissected from the surrounding fascia of the strap muscle (Fig. 2B). The resected specimen was extracted with a laparoscopic plastic bag through the central oral incision site. The oral vestibular port sites were closed with absorbable sutures. A drain was not placed.

**Figure 3:**
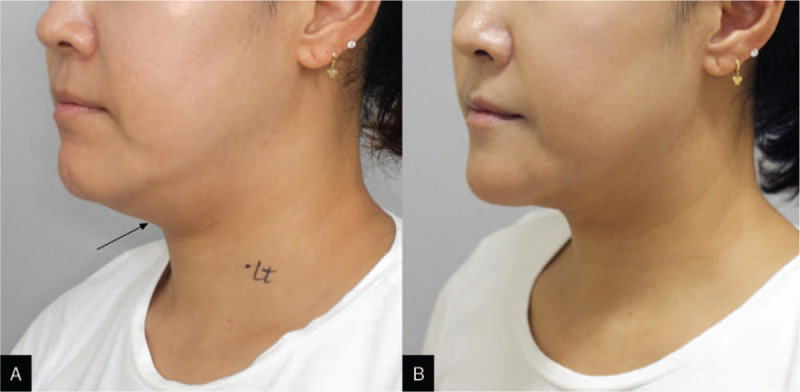
Excellent cosmetic outcome after endoscopic excision of a neck mass. (A) Preoperative state (arrow: mass). (B) Two months post-surgery.

We successfully completed the surgery without any complications. The operative time was 95.7 minutes. The patient was discharged on postoperative day 4. The final pathologic result was an epidermoid cyst. The patient was satisfied with her cosmetic outcome at 2 months post-surgery (Fig. 3A and B).

## 4 Discussion

Postoperative scars following neck mass removal significantly reduce the satisfaction of the operation for both the patient and the doctor. Moreover, it produces emotional stress, and correlates negatively with quality of life.^[11]^ Therefore, endoscopic or robot-assisted remote access surgery may be a suitable alternative to the traditional transcervical approach. In particular, the transoral vestibular approach has provided excellent cosmetic results, especially in the treatment of a thyroid mass, whether benign or malignant, without any external skin incision.^[6]^

Transoral endoscopic thyroidectomy was initially described in 2011 and has been popular since 2016.^[10,12,13]^ It is less invasive in terms of creating the working space than the transaxillary, breast, and postauricular approaches, as the extent of flap elevation and dissection is smaller. Transoral thyroidectomy was well indicated for selected patients with a thyroid mass, parathyroid tumor, and central neck dissection up to level VII in previous reports.^[2,14]^ There were also subsequent studies reporting that this procedure was shown to be feasible and safe, with surgical outcomes comparable to conventional thyroidectomy.^[15]^

We noted that we did not use this transoral approach in most upper neck mass cases which we treated. Therefore, we hypothesized that the transoral vestibular approach may be applied for the excision of an anterior upper neck mass on the extension of transoral thyroidectomy. It might be easier than thyroidectomy if the neck mass is located a shorter distance from the incision site and superficial area than the thyroid. In this study, the epidermoid cyst was located superficial to the fascia of the strap muscle. Therefore, it was easily removed with dissection of the surrounding soft tissue. It was a good opportunity for less experienced surgeons to familiarize themselves with this transoral approach.

However, some considerations must be taken into account to expand this procedure beyond thyroidectomy for other indications in the neck. The size of tumors may be one of the challenging factors, and can influence the surgical indication and limitation of the transoral approach, as the resected mass needs to be pulled out through a narrow transoral vestibular central incision site. If the neck mass is larger than 6 cm, it may be difficult to remove the lesion through the oral vestibular incision.^[13]^ In our case, the mass measured 2.4 cm, and it was easily removed through the transoral vestibular central incision site. In the case of a huge cystic mass, reducing the mass size in advance with needle aspiration may be considered, in order to decompress and decrease the tumor size.^[16]^

**Figure 4:**
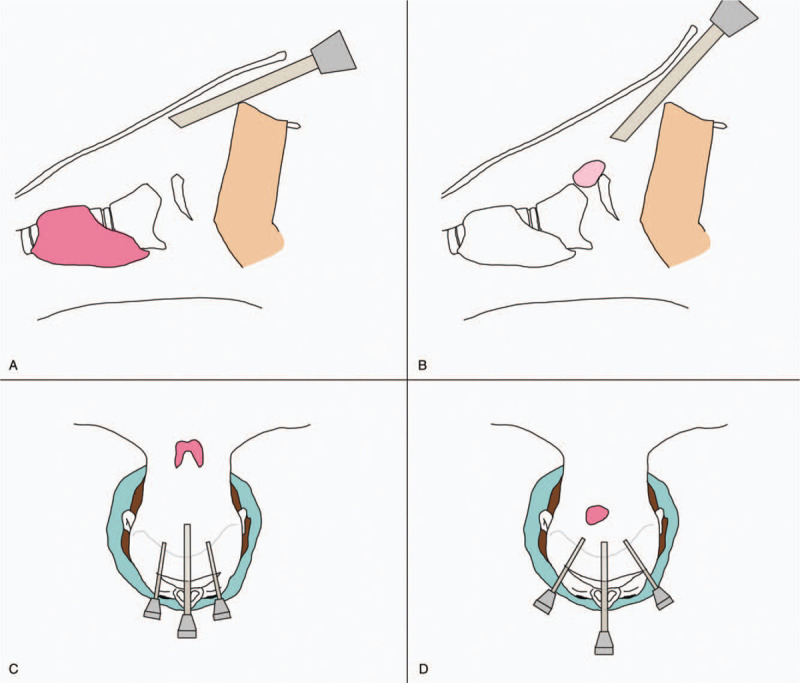
Schematic pictures for comparison of transoral vestibular approach for thyroid and upper neck mass. (A) and (C) for thyroid. (B) and (D) for upper neck mass.

The second consideration is the location of the mass. This case was more challenging using the transoral vestibular approach than with the lower neck mass, such as the thyroid, as the position of the mass was in the upper neck area. Because the mass was closer to the jaw, the angle between the trocars was larger during surgery. This required attention, because the mucosa of the median trocar area is torn further, and the oral commissure part of both lateral trocars is torn, and may remain as a scar after surgery (Fig. 4A–D). In this case, the right lip had a small tear, but it was later improved.

The final point to consider is any complication arising from the procedure. There are several previously reported complications related to the transoral approach, including mental nerve injury, infection of the surgical site, hematoma, subcutaneous emphysema, skin perforation, and CO_2_ embolism.^[6,17]^ Mental nerve injury is an important complication in transoral thyroidectomy. To prevent this, the lateral ports should be placed as close to the oral commissure as possible. This can prevent mental nerve injury, and also provide sufficient space and angle for handling the instruments with fewer collisions between the central endoscope and the 2 lateral robotic or endoscopic instruments. Moreover, we need to be aware of CO_2_ embolism when using CO_2_ gas. CO_2_ embolism is one of the fatal side effects for patients, and can occur when structures such as the anterior jugular veins are ruptured during blunt dissection, allowing CO_2_ to enter.^[18]^

This case is meaningful in that the transoral thyroidectomy vestibular approach is applied to the upper neck mass as it is, and it may contribute to the expansion of the indication of the vestibular incision approach. In addition, a further study with a larger number of cases and long-term follow-up is required in order to optimally evaluate the efficacy of this method.

In conclusion, endoscopic removal of a benign upper neck mass via a transoral vestibular approach can be useful for patients who wish to hide an anterior neck scar.

## Author contributions

**Conceptualization:** Dong Won Lee.

**Methodology:** Dong Won Lee, Kyung Tae.

**Writing – original draft:** Dong Won Lee.

**Writing – review & editing:** Dong Won Lee, Jeong Kyu Kim, Kyung Tae.

## Abbreviations

Abbreviations: CT = computed tomography, FNAB = fine needle aspiration biopsy, NOTES = natural orifice transluminal endoscopic surgery, US = ultrasound.
